# Sleep monitoring in ICU patients

**DOI:** 10.1186/cc14631

**Published:** 2015-03-16

**Authors:** S Namba, K Hayashi, T Hirayama, T Hirayama, Y Namba, M Terado, P Easton, Y Ujike

**Affiliations:** 1 Okayama University Hospital, Okayama, Japan; 2 University of Calgary, AB, Canada

## Introduction

Sleep disruption and deprivation is a continuing problem in the ICU. Strategies to improve sleep are confounded by difficulties in monitoring and measuring sleep in the ICU; traditional polysomnography cannot be utilized. Practical, non-intrusive diagnostic monitoring of sleep is required. The aims were to test two new ambulatory sleep diagnostic devices to monitor sleep in the ICU, compare sleep data generated by the different devices, and characterize sleep in the ICU.

## Methods

The devices were: Watch PAT 200 (Itamar), simple wristwatch style, employing peripheral arterial tone and actigraphy to evaluate sleep time and sleep stage by an automatic algorithm (PAT device); and ALICE PDx (Respironics Philips), miniature polysomnographic device utilizing EEG and EMG recordings, requiring post-study sleep technician scoring (PSG device). Nineteen ICU patients provided informed consent (mean age 37 years, two female). Diagnosis of most patients was trauma. Device technical problems terminated one ALICE PDx study and three Watch PAT study; one patient revoked consent. Therefore, 14 patients were recorded successfully in a private room in the ICU, while simultaneously wearing both devices, from 20:00 to 06:00. No patient received sedation. Subjective sleep quality was estimated by the visual analog scale.

## Results

Both devices calculated total sleep time (TST), but the results were significantly different (*P *< 0.05), with mean TST reported as 443.07 and 270.8 minutes for PAT and PSG devices. VAS correlated tightly with TST calculated by the PSG device (*r *= 0.559, *P *< 0.05). Both devices were able to successfully discern different sleep stages, summarized as light sleep, deep sleep, and REM. Measurements of sleep stage were generally in agreement between the two devices; REM sleep time correlated strongly between PAT and PSG devices (*P *< 0.05). Sleep stage distribution was light sleep 62.2% (PAT) and 74.1% (PSG); REM 13.0% (PAT) and 10.7% (PSG); deep sleep 14.5% (PAT) and 7.9% (PSG).

## Conclusion

Both wristwatch-style PAT and miniature PSG devices successfully recorded sleep in ICU patients. Although the simple PAT device overestimated TST, sleep stage times were generally in agreement, especially REM time which correlated strongly. Both devices can be used to effectively monitor and characterize sleep in the ICU environment.

